# Supplementary material for: The Norwegian version of the Copenhagen Psychosocial Questionnaire (COPSOQ III): Initial validation study using a national sample of registered nurses
Source: PLoS One. 2023 Aug 24;18(8):e0289739. doi: 10.1371/journal.pone.0289739 (PMC10449149; doi:10.1371/journal.pone.0289739)
Supplement: S1 Appendix — (DOCX) [file pone.0289739.s001.docx]

# S1 Appendix. The translated items.

| Dimension  English/Norwegian | Dimension | Item name | Level | Question | Item in Norwegian  Black font: Included in the Norwegian version; Grey font: Not included | Response options |
| --- | --- | --- | --- | --- | --- | --- |
| Quantitative Demands/  Kvantitative krav | QD | QD1 | MIDDLE | Is your workload unevenly distributed so it piles up? | Er arbeidsmengden din ujevnt fordelt slik at arbeid hoper seg opp? | 1 |
|  |  | QD2 | CORE | How often do you not have time to complete all your work tasks? | Hvor ofte har du ikke tid til å fullføre alle arbeidsoppgavene dine? | 1 |
|  |  | QD3 | CORE | Do you get behind with your work? | Blir du hengende etter med arbeidsoppgavene dine? | 1 |
|  |  | QD4 | LONG | Do you have enough time for your work tasks? | Har du nok tid til arbeidsoppgavene dine? | 1R |
| Work Pace/  Arbeidstempo | WP | WP1 | CORE | Do you have to work very fast? | Må du arbeide veldig raskt? | 1 |
|  |  | WP2 | CORE | Do you work at a high pace throughout the day? | Arbeider du i et høyt tempo gjennom hele dagen? | 2 |
|  |  | WP3 | LONG | Is it necessary to keep working at a high pace? | Er det nødvendig å stadig jobbe i et høyt tempo? | 2 |
| Cognitive Demands/  Kognitive krav | CD | CD1 | LONG | Do you have to keep your eyes on lots of things while you work? | Må du følge med på mange ting mens du arbeider? | 1 |
|  |  | CD2 | LONG | Does your work require that you remember a lot of things? | Krever arbeidet ditt at du må huske på mange ting? | 1 |
|  |  | CD3 | LONG | Does your work demand that you are good at coming up with new ideas? | Krever arbeidet ditt at du er flink til å komme opp med nye idéer? | 1 |
|  |  | CD4 | LONG | Does your work require you to make difficult decisions? | Krever arbeidet ditt at du må ta vanskelige beslutninger? | 1 |
| Emotional Demands/  Emosjonelle krav | ED | ED1 | MIDDLE | Does your work put you in emotionally disturbing situations? | Setter arbeidet ditt deg i følelsesmessig krevende situasjoner? | 1 |
|  |  | EDX2 | CORE | Do you have to deal with other people’s personal problems as part of your work? | Må du håndtere andre folks personlige problemer som del av jobben din? | 1 |
|  |  | ED3 | CORE | Is your work emotionally demanding? | Er arbeidet ditt følelsesmessig krevende? | 2 |
| Demands for Hiding Emotions/  Krav om å skjule følelser | HE | HE1 | LONG | Are you required to treat everyone equally, even if you do not feel like it? | Må du behandle alle likt selv om du ikke føler for det? | 1 |
|  |  | HE2 | MIDDLE | Does your work require that you hide your feelings? | Krever arbeidet ditt at du skjuler følelsene dine? | 2 |
|  |  | HE3 | MIDDLE | Are you required to be kind and open towards everyone – regardless of how they behave towards you? | Må du være vennlig og åpen mot alle - uavhengig av hvordan de oppfører seg mot deg? | 2 |
|  |  | HE4 | MIDDLE | Does your work require that you do not state your opinion? | Krever arbeidet ditt at du ikke uttrykker din egen mening? | 1 |
| Influence at Work/  Innflytelse på jobben | IN | INX1 | CORE | Do you have a large degree of influence on the decisions concerning your work? | Har du mulighet til å påvirke vesentlige beslutninger som angår arbeidet ditt? | 1 |
|  |  | IN2 | LONG | Do you have a say in choosing who you work with? | Kan du påvirke hvem du arbeider sammen med? | 1 |
|  |  | IN3 | MIDDLE | Can you influence the amount of work assigned to you? | Kan du påvirke arbeidsmengden din? | 1 |
|  |  | IN4 | MIDDLE | Do you have any influence on what you do at work? | Kan du påvirke hvilke arbeidsoppgaver du gjør på jobb? | 1 |
|  |  | IN5 | LONG | Can you influence how quickly you work? | Kan du påvirke ditt arbeidstempo? | 1 |
|  |  | IN6 | MIDDLE | Do you have any influence on HOW you do your work? | Har du innflytelse på HVORDAN du utfører arbeidsoppgavene dine? | 1 |
| Possibilities for Development/  Muligheter for utvikling | PD | PD2 | CORE | Do you have the possibility of learning new things through your work? | Har du mulighet til å lære deg noe nytt gjennom arbeidet ditt? | 2 |
|  |  | PD3 | CORE | Can you use your skills or expertise in your work? | Kan du bruke ferdighetene dine eller kompetansen din i arbeidet? | 2 |
|  |  | PD4 | MIDDLE | Does your work give you the opportunity to develop your skills? | Får du muligheten til å utvikle ferdighetene dine gjennom jobben? | 2 |
| Variation of Work/  Variasjon i arbeidet | VA | VA1 | LONG | Is your work varied? | Er arbeidet ditt variert? | 1 |
|  |  | VA2 | LONG | Do you have to do the same thing over and over again? | Må du gjøre det samme om og om igjen? | 1R |
| Control over Working Time/ Kontroll over arbeidstid | CT | CT1 | MIDDLE | Can you decide when to take a break? | Kan du bestemme når du tar pause? | 1 |
|  |  | CT2 | MIDDLE | Can you take holidays more or less when you wish? | Kan du stort sett ta ferie når du ønsker det? | 1 |
|  |  | CT3 | MIDDLE | Can you leave your work to have a chat with a colleague? | Kan du forlate arbeidsoppgavene dine for å prate med en kollega? | 1 |
|  |  | CT4 | MIDDLE | If you have some private business, is it possible for you to leave your place of work for half an hour without special permission? | Hvis du har private ærender, kan du forlate arbeidsplassen i en halvtime uten å be om tillatelse? | 1 |
|  |  | CT5 | LONG | Do you have to do overtime? | Må du arbeide overtid? | 1R |
| Meaning of Work/  Arbeidets betydning | MW | MW1 | CORE | Is your work meaningful? | Er arbeidet ditt meningsfylt? | 2 |
|  |  | MW2 | MIDDLE | Do you feel that the work you do is important? | Føler du at arbeidet du gjør er viktig? | 2 |
| Predictability/  Forutsigbarhet | PR | PR1 | CORE | At your place of work, are you informed well in advance concerning, for example, important decisions, changes or plans for the future? | Får du informasjon i god tid når det gjelder f.eks. viktige beslutninger, endringer eller planer for framtiden på arbeidsplassen din? | 2 |
|  |  | PR2 | CORE | Do you receive all the information you need in order to do your work well? | Får du all den informasjonen du trenger for å gjøre en god jobb? | 2 |
| Recognition/  Anerkjennelse | RE | RE1 | CORE | Is your work recognized and appreciated by the management? | Blir arbeidet ditt anerkjent og verdsatt av ledelsen? | 2 |
|  |  | RE2 | LONG | Does the management at your workplace respect you? | Respekterer ledelsen på din arbeidsplass deg? | 2 |
|  |  | RE3 | LONG | Are you treated fairly at your workplace? | Blir du behandlet rettferdig på arbeidsplassen din? | 2 |
| Role Clarity/  Rolleklarhet | CL | CL1 | CORE | Does your work have clear objectives? | Er det klare mål for arbeidet ditt? | 2 |
|  |  | CL2 | MIDDLE | Do you know exactly which areas are your responsibility? | Vet du nøyaktig hvilke ansvarsområder du har? | 2 |
|  |  | CL3 | MIDDLE | Do you know exactly what is expected of you at work? | Vet du nøyaktig hva som forventes av deg på jobb? | 2 |
| Role Conflicts/  Rollekonflikter | CO | CO2 | CORE | Are contradictory demands placed on you at work? | Får du motstridende krav på jobb? | 2 |
|  |  | CO3 | CORE | Do you sometimes have to do things that ought to have been done in a different way? | Må du noen ganger gjøre ting som burde vært gjort på en annen måte? | 2 |
| Illegitimate Tasks/  Unødvendige oppgaver | IT | IT1 | MIDDLE | Do you sometimes have to do things that seem to be unnecessary? | Må du noen ganger gjøre ting som virker unødvendig? | 2 |
| Quality of Leadership/  Ledelseskvalitet | QL | QL_T |  | To what extent would you say that your immediate superior ... | I hvilken grad vil du si at din nærmeste leder: |  |
|  |  | QLX1 | MIDDLE | - makes sure that the members of staff have good development opportunities? | - sørger for at alle ansatte har gode utviklingsmuligheter? | 2† |
|  |  | QL2 | LONG | - gives high priority to job satisfaction? | - prioriterer jobbtilfredshet høyt? | 2† |
|  |  | QL3 | CORE | - is good at work planning? | - er god til å planlegge arbeidet? | 2† |
|  |  | QL4 | CORE | - is good at solving conflicts? | - er god til å håndtere konflikter? | 2† |
| Social Support from Supervisor/  Sosial støtte fra overordnet | SS | SSX1 | MIDDLE | How often is your immediate superior willing to listen to your problems at work, if needed? | Hvor ofte er din nærmeste leder villig til å lytte til de jobbrelaterte problemene dine, om det trengs? | 1† |
|  |  | SSX2 | CORE | How often do you get help and support from your immediate superior, if needed? | Hvor ofte får du hjelp og støtte fra din nærmeste leder, om det trengs? | 1† |
|  |  | SSX3 | LONG | How often does your immediate superior talk with you about how well you carry out your work? | Hvor ofte snakker din nærmeste leder med deg om hvor godt du utfører arbeidet ditt? | 1† |
| Social Support from Colleagues/  Sosial støtte fra kolleger | SC | SCX1 | CORE | How often do you get help and support from your colleagues, if needed? | Hvor ofte får du hjelp og støtte fra dine kolleger, om det trengs? | 1‡ |
|  |  | SCX2 | MIDDLE | How often are your colleagues willing to listen to your problems at work, if needed? | Hvor ofte er dine kolleger villig til å lytte til de jobbrelaterte problemene dine, om det trengs? | 1‡ |
|  |  | SCX3 | LONG | How often do your colleagues talk with you about how well you carry out your work? | Hvor ofte snakker dine kolleger med deg om hvor godt du utfører arbeidet ditt? | 1‡ |
| Sense of Community at Work/  Opplevelse av samhold på jobb | SW | SW1 | CORE | Is there a good atmosphere between you and your colleagues? | Er det god stemning mellom deg og dine kolleger? | 1‡ |
|  |  | SW2 | LONG | Is there good co-operation between the colleagues at work? | Er det godt samarbeid mellom kollegene på arbeidsplassen din? | 1‡ |
|  |  | SW3 | MIDDLE | Do you feel part of a community at your place of work? | Føler du deg som en del av et fellesskap på arbeidsplassen din? | 1‡ |
| Commitment to the Workplace/Forpliktelse til arbeidsplassen | CW | CW1 | LONG | Do you enjoy telling others about your place of work? | Liker du å fortelle andre om arbeidsplassen din? | 2 |
|  |  | CW2 | LONG | Do you feel that your place of work is of great importance to you? | Føler du arbeidsplassen din har stor betydning for deg? | 2 |
|  |  | CWX3 | LONG | Would you recommend other people to apply for a position at your workplace? | Ville du anbefalt andre å søke jobb på arbeidsplassen din? | 2 |
|  |  | CW4 | LONG | How often do you consider looking for work elsewhere? | Hvor ofte vurderer du å se etter jobb andre steder? | 1R |
|  |  | CW5 | LONG | Are you proud of being part of this organization? | Er du stolt av å være en del av organisasjonen du arbeider i? | 2 |
| Work Engagement/  Arbeids-engasjement | WE | WE_T |  | How often do you experience the following? | Hvor ofte opplever du følgende: |  |
|  |  | WE1 | LONG | At my work, I feel bursting with energy. | På jobb føler jeg meg full av energi. | 3 |
|  |  | WE2 | LONG | I am enthusiastic about my job. | Jeg er sterkt engasjert i jobben min. | 3 |
|  |  | WE3 | LONG | I am immersed in my work. | Jeg blir oppslukt i arbeidet mitt. | 3 |
| Job Insecurity/  Jobbusikkerhet | JI | JI1 | CORE | Are you worried about becoming unemployed? | Er du bekymret for å bli arbeidsledig? | 2 |
|  |  | JI2 | LONG | Are you worried about new technology making you redundant? | Er du bekymret for at ny teknologi skal gjøre deg overflødig? | 2 |
|  |  | JI3 | CORE | Are you worried about it being difficult for you to find another job if you became unemployed? | Er du bekymret for at det vil være vanskelig for deg å finne en annen jobb om du skulle bli arbeidsledig? | 2 |
| Insecurity over Working Conditions/  Usikkerhet rundt arbeidsbetingelser | IW | IW1 | CORE | Are you worried about being transferred to another job against your will? | Er du bekymret for å bli omplassert mot din vilje? | 2 |
|  |  | IW2 | LONG | Are you worried about your working tasks being changed against your will? | Er du bekymret for at arbeidsoppgavene dine skal bli endret mot din vilje? | 2 |
|  |  | IW3 | MIDDLE | Are you worried about the timetable being changed (shift, weekdays, time to enter and leave ...) against your will? | Er du bekymret for at arbeidstiden din endres (skift/vakter, dager, tidspunkt for start og avslutning?) mot din vilje? | 2 |
|  |  | IW4 | MIDDLE | Are you worried about a decrease in your salary (reduction, variable pay being introduced ...)? | Er du bekymret for nedgang i lønn (reduksjon, innføring av variabel lønn, tap av tillegg etc ...)? | 2 |
|  |  | IW5 | LONG | Are there good prospects in your job? | Er det gode framtidsutsikter i jobben din? | 2 |
| Quality of Work/  Kvalitet på arbeidet | QW | QW1 | LONG | To what extent do you find it possible to perform your work tasks at a satisfactory quality? | I hvilken grad synes du det er mulig å utføre arbeidsoppgavene dine med tilfredsstillende kvalitet? | 2 |
|  |  | QW2 | MIDDLE | Are you satisfied with the quality of the work performed at your workplace? | Er du fornøyd med kvaliteten på arbeidet som utføres på arbeidsplassen din? | 2 |
| Job Satisfaction/  Jobbtilfredshet | JS | JS_T |  | Regarding your work in general, how pleased are you with: | Hvor fornøyd er du med: |  |
|  |  | JS1 | MIDDLE | - your work prospects? | - fremtidsutsiktene dine i jobben? | 6 |
|  |  | JS2 | LONG | - the physical working conditions? | - de fysiske arbeidsforholdene? | 6 |
|  |  | JS3 | LONG | - the way your abilities are used? | - måten kunnskapen og evnene dine benyttes? | 6 |
|  |  | JS4 | CORE | - your job as a whole, everything taken into consideration? | - jobben som helhet, alt tatt i betraktning? | 6 |
|  |  | JS5 | MIDDLE | - your salary? | - lønna di? | 6 |
| Work–Life Conflict/  Arbeid-fritid konflikt | WF | WF_T |  | The next five questions concern the ways in which your work affects your private life: | De fem neste spørsmålene angår måten arbeidet ditt påvirker privatlivet. |  |
|  |  | WFX1 | LONG | Are there times when you need to be at work and at home at the same time? | Hender det at du trenger å være på jobb og hjemme samtidig? | 1 |
|  |  | WF2 | CORE | Do you feel that your work drains so much of your energy that it has a negative effect on your private life? | Føler du at arbeidet ditt tar så mye energi at det har negativ påvirkning på privatlivet ditt? | 2 |
|  |  | WF3 | CORE | Do you feel that your work takes so much of your time that it has a negative effect on your private life? | Opplever du at arbeidet ditt tar så mye av tiden din at det har en negativ effekt på privatlivet ditt? | 2 |
|  |  | WF5 | LONG | The demands of my work interfere with my private and family life? | Opplever du at kravene på jobb forstyrrer privatlivet ditt? | 2 |
|  |  | WF6 | LONG | Due to work-related duties, I have to make changes to my plans for private and family activities. | Jeg må endre planene mine for privat- og familieaktiviteter på grunn av arbeidsrelaterte forpliktelser. | 2 |
| (Intro Trust & Justice)/(Intro til tillit og rettferdighet) |  |  |  | The next questions are not about your own job but about the workplace as a whole. | De neste spørsmålene er ikke om din jobb, men om arbeidsplassen som helhet. |  |
| Horizontal Trust/  Horisontal tillit | TE | TE1 | LONG | Do the employees withhold information from each other? | Holder de ansatte tilbake informasjon fra hverandre? | 2R |
|  |  | TE2 | LONG | Do the employees withhold information from the management? | Holder de ansatte tilbake informasjon fra ledelsen? | 2R |
|  |  | TE3 | MIDDLE | Do the employees in general trust each other? | Stoler de ansatte generelt på hverandre? | 2 |
| Vertical Trust/  Vertikal tillit | TM | TM1 | CORE | Does the management trust the employees to do their work well? | Stoler ledelsen på at de ansatte gjør en god jobb? | 2 |
|  |  | TMX2 | CORE | Can the employees trust the information that comes from the management? | Kan de ansatte stole på informasjonen som kommer fra ledelsen? | 2 |
|  |  | TM3 | LONG | Does the management withhold important information from the employees? | Holder ledelsen tilbake viktig informasjon fra de ansatte? | 2R |
|  |  | TM4 | MIDDLE | Are the employees able to express their views and feelings? | Er det mulig for ansatte å uttrykke sine synspunkt og følelser? | 2 |
| Organizational Justice/  Organisasjons-rettferdighet | JU | JU1 | CORE | Are conflicts resolved in a fair way? | Løses konflikter på en rettferdig måte? | 2 |
|  |  | JU2 | LONG | Are employees appreciated when they have done a good job? | Blir ansatte satt pris på når de har gjort en god jobb? | 2 |
|  |  | JU3 | LONG | Are all suggestions from employees treated seriously by the management? | Blir alle forslag fra ansatte behandlet seriøst av ledelsen? | 2 |
|  |  | JU4 | CORE | Is the work distributed fairly? | Er arbeidsoppgavene rettferdig fordelt? | 2 |
| (Intro Negative Acts)/  (Intro negative handlinger) |  |  |  | Conflicts and offensive behaviours | Konflikter og uønsket adferd |  |
| Gossip and Slander/  Sladder og baksnakking | GS | GS1 | LONG | Have you been exposed to gossip and slander at your workplace during the last 12 months? | Har du blitt utsatt for sladder og baksnakking på din arbeidsplass i løpet av de siste 12 månedene? | 4 |
|  |  | GS2 | LONG | If yes, from whom? (You may tick more than one) | Hvis ja, fra hvem? (det er mulig å sette flere kryss) | 5M |
| Conflicts and Quarrels/  Konflikter og krangler | CQ | CQ1 | LONG | Have you been involved in quarrels or conflicts at your workplace during the last 12 months? | Har du vært involvert i krangler eller konflikter på arbeidsplassen din de siste 12 månedene? | 4 |
| Unpleasant Teasing/  Ubehagelig erting | UT | UT1 | LONG | Have you been exposed to unpleasant teasing at your workplace during the last 12 months? | Har du blitt utsatt for ubehagelig erting på arbeidsplassen din de siste 12 månedene? | 4 |
|  |  | UT2 | LONG | If yes, from whom? (You may tick more than one) | Hvis ja, fra hvem? (det er mulig å sette flere kryss) | 5M |
| Cyberbullying/  Nettmobbing | HSM | HSM1 | LONG | Have you been exposed to work-related harassment on social media (e.g., Facebook), by e-mail or text messages during the last 12 months? | Har du blitt utsatt for arbeidsrelatert trakassering på sosiale media, e-post eller tekstmeldinger de siste 12 månedene? | 4 |
|  |  | HSM2 | LONG | If yes, from whom? (You may tick more than one) | Hvis ja, fra hvem? (det er mulig å sette flere kryss) | 5M |
| Sexual Harassment/  Seksuell trakassering | SH | SH1 | LONG | Have you been exposed to undesired sexual attention at your workplace during the last 12 months? | Har du blitt utsatt for uønsket seksuell oppmerksomhet på arbeidsplassen din de siste 12 månedene? | 4 |
|  |  | SH2 | LONG | If yes, from whom? (You may tick more than one) | Hvis ja, fra hvem? (det er mulig å sette flere kryss) | 5M |
| Threats of Violence/  Voldstrusler | TV | TV1 | LONG | Have you been exposed to threats of violence at your workplace during the last 12 months? | Har du blitt utsatt for trusler om vold på arbeidsplassen din de siste 12 månedene? | 4 |
|  |  | TV2 | LONG | If yes, from whom? (You may tick more than one) | Hvis ja, fra hvem? (det er mulig å sette flere kryss) | 5M |
| Physical Violence/  Fysisk vold | PV | PV1 | LONG | Have you been exposed to physical violence at your workplace during the last 12 months? | Har du blitt utsatt for fysisk vold på arbeidsplassen din de siste 12 månedene? | 4 |
|  |  | PV2 | LONG | If yes, from whom? (You may tick more than one) | Hvis ja, fra hvem? (det er mulig å sette flere kryss) | 5M |
| Bullying/  Mobbing | BU | BU1 | LONG | Bullying means that a person repeatedly is exposed to unpleasant or degrading treatment, and that the person finds it difficult to defend himself or herself against it. Have you been exposed to bullying at your workplace during the last 12 months? | Mobbing betyr at en person gjentatte ganger blir utsatt for ubehagelig eller nedverdigende behandling, og at personen finner det vanskelig å forsvare seg mot dette. Har du blitt utsatt for mobbing på arbeidsplassen din i løpet av de siste 12 månedene? | 4 |
|  |  | BU3 | LONG | If yes, from whom? (You may tick more than one) | Hvis ja, fra hvem? (det er mulig å sette flere kryss) | 5M |
|  |  | BU2 | LONG | How often do you feel unjustly criticized, bullied or shown up in front of others by your colleagues or your superior? | Hvor ofte føler du deg urettmessig kritisert, mobbet eller ydmyket foran andre, av dine kolleger eller din leder? | 1§ |
| (Intro Health)/  (Intro helse) |  |  |  | The following questions are about your own health and well-being. Please do not try to distinguish between symptoms that are caused by work and symptoms that are due to other causes. The task is to describe how you are in general. | De følgende spørsmålene angår din helse og trivsel. Vennligst ikke forsøk å skille mellom symptomer som skyldes arbeid og symptomer som skyldes andre årsaker. Oppgaven er å beskrive hvordan du har det generelt. |  |
|  |  |  |  | The questions are about your health and well-being during the last 4 weeks. | Disse spørsmålene gjelder din helse og trivsel de siste 4 ukene. |  |
| Self-Rated Health/  Egenvurdert helse | GH | GH1 | CORE | In general, would you say your health is: | Generelt, vil du si at din helse er: | 7 |
|  |  | GH2 | LONG | If you evaluate the best conceivable state of health at 10 points and the worst at 0 points, how many points do you then give your present state of health? | Hvis du tenker deg den best tenkelige helsetilstanden på 10 poeng, og den verst tenkelige på 0: hvor mange poeng vil du gi din nåværende helsetilstand? | 8 |
| Sleeping Troubles/  Søvnproblemer | SL | SL_T | LONG | These questions are about how you have been during the last 4 weeks. | Disse spørsmålene handler om hvordan du har hatt det de siste 4 ukene. |  |
|  |  | SL1 | LONG | How often have you slept badly and restlessly? | Hvor ofte har du sovet dårlig eller urolig? | 9 |
|  |  | SL2 | LONG | How often have you found it hard to go to sleep? | Hvor ofte har du hatt vansker med å sovne? | 9 |
|  |  | SL3 | LONG | How often have you woken up too early and not been able to get back to sleep? | Hvor ofte har du våknet opp for tidlig og ikke klart å sovne igjen? | 9 |
|  |  | SL4 | LONG | How often have you woken up several times and found it difficult to get back to sleep? | Hvor ofte har du våknet opp flere ganger og hatt vansker med å sovne igjen? | 9 |
| Burnout/  Utbrenthet | BO | BO_T | LONG | These questions are about how you have been during the last 4 weeks. | Disse spørsmålene handler om hvordan du har hatt det de siste 4 ukene. |  |
|  |  | BO1 | LONG | How often have you felt worn-out? | Hvor ofte har du følt deg utslitt? | 9 |
|  |  | BO2 | LONG | How often have you been physically exhausted? | Hvor ofte har du vært fysisk utmattet? | 9 |
|  |  | BO3 | LONG | How often have you been emotionally exhausted? | Hvor ofte har du vært følelsesmessig utmattet? | 9 |
|  |  | BO4 | LONG | How often have you felt tired? | Hvor ofte har du følt deg trøtt? | 9 |
| Stress/  Stress | ST | ST_T | LONG | These questions are about how you have been during the last 4 weeks. | Disse spørsmålene handler om hvordan du har hatt det de siste 4 ukene. |  |
|  |  | ST1 | LONG | How often have you had problems relaxing? | Hvor ofte har du hatt problemer med å slappe av? | 9 |
|  |  | ST2 | LONG | How often have you been irritable? | Hvor ofte har du vært irritabel? | 9 |
|  |  | ST3 | LONG | How often have you been tense? | Hvor ofte har du vært anspent? | 9 |
| Somatic Stress/  Somatisk/kroppslig stress | SO | SO_T | LONG | These questions are about how you have been during the last 4 weeks. | Disse spørsmålene handler om hvordan du har hatt det de siste 4 ukene. |  |
|  |  | SO1 | LONG | How often have you had stomach-ache? | Hvor ofte har du hatt vondt i magen? | 9 |
|  |  | SO2 | LONG | How often have you had a headache? | Hvor ofte har du hatt vondt i hodet? | 9 |
|  |  | SO3 | LONG | How often have you had palpitations? | Hvor ofte har du hatt hjertebank? | 9 |
|  |  | SO4 | LONG | How often have you had tension in various muscles? | Hvor ofte har du hatt muskel- og skjelettplager? | 9 |
| Cognitive Stress/  Kognitivt stress | CS | CS_T | LONG | These questions are about how you have been during the last 4 weeks. | Disse spørsmålene handler om hvordan du har hatt det de siste 4 ukene. |  |
|  |  | CS1 | LONG | How often have you had problems concentrating? | Hvor ofte har du hatt problemer med å konsentrere deg? | 9 |
|  |  | CS2 | LONG | How often have you found it difficult to think clearly? | Hvor ofte har du hatt vansker med å tenke klart? | 9 |
|  |  | CS3 | LONG | How often have you had difficulty in taking decisions? | Hvor ofte har du hatt vansker med å ta beslutninger? | 9 |
|  |  | CS4 | LONG | How often have you had difficulty with remembering? | Hvor ofte har du hatt vansker med å huske? | 9 |
| Depressive Symptoms/  Depressive symptomer | DS | DS_T | LONG | These questions are about how you have been during the last 4 weeks. | Disse spørsmålene handler om hvordan du har hatt det de siste 4 ukene. |  |
|  |  | DS1 | LONG | How often have you felt sad? | Hvor ofte har du følt deg trist? | 9 |
|  |  | DS2 | LONG | How often have you lacked self-confidence? | Hvor ofte har du manglet selvtillit? | 9 |
|  |  | DS3 | LONG | How often have you had a bad conscience or felt guilty? | Hvor ofte har du hatt dårlig samvittighet eller hatt skyldfølelse? | 9 |
|  |  | DS4 | LONG | How often have you lacked interest in everyday things? | Hvor ofte har du manglet interesse for hverdagslige ting? | 9 |
| Self-Efficacy/  Mestringstro | SE | SE_T |  | How well do these descriptions fit you as a person? | Hvor godt passer disse beskrivelsene deg som person? |  |
|  |  | SE1 | LONG | I am always able to solve difficult problems, if I try hard enough. | Jeg er alltid i stand til å løse vanskelige problemer om jeg prøver hardt nok. | 10 |
|  |  | SE2 | LONG | If people work against me, I find a way of achieving what I want. | Hvis folk motarbeider meg, finner jeg måter å oppnå det jeg vil. | 10 |
|  |  | SE3 | LONG | It is easy for me to stick to my plans and achieve my objectives. | Det er lett for meg å holde meg til planene mine og oppnå målene mine. | 10 |
|  |  | SE4 | LONG | I feel confident that I can handle unexpected events. | Jeg føler meg trygg på at jeg kan håndtere uventede hendelser. | 10 |
|  |  | SE5 | LONG | When I have a problem, I can usually find several ways of solving it. | Når jeg har et problem, kan jeg vanligvis finne flere måter å løse det på. | 10 |
|  |  | SE6 | LONG | Regardless of what happens, I usually manage. | Som regel klarer jeg meg uansett hva som skjer. | 10 |

Note that CORE items are mandatory in all short, middle, and long national versions of COPSOQ. The choice of items for national MIDDLE versions can deviate from the international version listed here.

*Explanation of response options (and values for the scale; each scale is scored in the direction indicated by the scale name):

1: Always (100); Often (75); Sometimes (50); Seldom (25); Never/hardly ever (0) 1R: Always (0); Often (25); Sometimes (50); Seldom (75); Never/hardly ever (100) (Reverse scoring)

2: To a very large extent (100); To a large extent (75); Somewhat (50); To a small extent (25); To a very small extent (0) 2R: To a very large extent (0); To a large extent (25); Somewhat (50); To a small extent (25); To a very small extent (100) (Reverse scoring)

3: Never (0), Seldom (25), Sometimes (50), Often (75), Always (100)

4: Yes, daily; Yes, weekly; Yes, monthly; Yes, a few times; No

5M: Colleagues, Manager/superior, Subordinates, Clients/customers/patients (Multiple response options)

6: Very satisfied (100), Satisfied (75), Neither/Nor (50), Unsatisfied (25), Very unsatisfied (0)

7: Excellent (100), Very good (75), Good (50), Fair (25), Poor (0) 8: 0, 1, 2, 4, 5, 6, 7, 8, 9, 10

9: All the time (100); A large part of the time (75); Part of the time (50); A small part of the time (25); Not at all (0)

10: Fits perfectly (100); Fits quite well (67); Fits a little bit (33); Does not fit (0)

†Including the response option, if deemed necessary: ‘I do not have a supervisor’ (coded as missing).

‡Including the response option, if deemed necessary: ‘I do not have colleagues’ (coded as missing).

§ Including the response option, if deemed necessary: ‘I do not have a superior/colleagues’ (coded as missing). Source: Schaufeli WB, Bakker AB, Salanova M. The measurement of work engagement with a short questionnaire. Educ Psychol Meas. 2006; 66:701-716.

Merk: Core elementene/items er obligatorisk i alle korte, middels og lange nasjonale versjoner av COPSOQ. Valg av items for den nasjonale MIDDEL versjonen kan avvike fra den internasjonale versjonen som er listet opp her. *Forklaring på svaralternativene (og verdiene for skalaer - hver skala er skåret i retningen som er indikert av skala navnet):

1: Alltid (100); Ofte (75); Av og til (50); Sjelden (25); Aldri/nesten aldri (0)

1R: Alltid (0); Ofte (25); Av og til (50); Sjelden (75): Aldri/nesten aldri (100) (Reversert skåring)

2: I svært stor grad (100); I stor grad (75); I noen grad (50); I lite grad (25); I svært liten grad (0)

2R: I svært stor grad (0); I stor grad (25); I noen grad (50); I liten grad (75); I svært liten grad (100) (Reversert skåring)

3: Aldri (0); Sjelden (25); Noen ganger (50); Ofte (75); Alltid (100) 4: Ja, daglig; Ja, ukentlig; Ja, månedlig; Ja, noen få ganger; Nei

5M: Kolleger, Leder/overordnet, Underordnet, Klienter/kunder/pasienter (flere svaralternativ)

6: Svært tilfreds (100), Tilfreds (75), Verken/eller (50), Utilfreds (25), Svært utilfreds (0)

7: Fortreffelig (100); Svært god/godt (75); God/godt (50); Ikke helt god/godt (25); Dårlig (0)

8: 0, 1, 2, 4, 5, 6, 7, 8, 9, 10

9: Hele tiden (100): En stor del av tiden (75); Deler av tiden (50); En liten del av tiden (25); Ikke i det hele tatt (0)

10: Passer perfekt (100); Passer ganske bra (67); Passer litt (33); Passer ikke (0)

†Inkluderer svaralternativet om det vurderes som nødvendig: 'Jeg har ikke noen leder' (kodet som missing).

‡ Inkluderer svaralternativet om det vurderes som nødvendig: 'Jeg har ingen kolleger (kodet som missing).

§ Inkluderer svaralternativet om det vurderes som nødvendig: 'Jeg har ingen leder / kolleger (kodet som missing). Kilde: Schaufeli WB, Bakker AB, Salanova M. The measurement of work engagement with a short questionnaire. Educational and Psychological Measurement. 2006; 66:701-716.
