# Supplementary material for: The Norwegian version of the Copenhagen Psychosocial Questionnaire (COPSOQ III): Initial validation study using a national sample of registered nurses
Source: PLoS One. 2023 Aug 24;18(8):e0289739. doi: 10.1371/journal.pone.0289739 (PMC10449149; doi:10.1371/journal.pone.0289739)
Supplement: S2 Appendix — S1 Table. Kaiser–Meyer–Olkin measure of sampling adequacy. S2 Table. Confirmatory factor analysis. S3 Table. Exploratory factor analysis, maximum likelihood and factor loadings. (DOCX) [file pone.0289739.s002.docx]

# S2 Appendix. Results from factor analyses.

S1 Table. Kaiser–Meyer–Olkin measure of sampling adequacy.

| Variable | Kaiser–Meyer–Olkin measure of sampling adequacy |
| --- | --- |
| QD1 | 0.9687 |
| QD2 | 0.9395 |
| QD3 | 0.9329 |
| WP1 | 0.9429 |
| WP2 | 0.9277 |
| CD1 | 0.9233 |
| CD2 | 0.9134 |
| CD3 | 0.888 |
| CD4 | 0.948 |
| ED1 | 0.9097 |
| EDX2 | 0.9294 |
| ED3 | 0.8994 |
| HE1 | 0.899 |
| HE2 | 0.9636 |
| HE3 | 0.9271 |
| HE4 | 0.9568 |
| INX1 | 0.9663 |
| IN2 | 0.9609 |
| IN3 | 0.96 |
| IN4 | 0.9477 |
| PD2 | 0.9545 |
| PD3 | 0.9657 |
| PD4 | 0.9665 |
| VA1 | 0.945 |
| VA2r | 0.9403 |
| CT1 | 0.9593 |
| CT2 | 0.9736 |
| CT3 | 0.9524 |
| CT4 | 0.9289 |
| CT5r | 0.9732 |
| MW1 | 0.9627 |
| MW2 | 0.9515 |
| PR1 | 0.9864 |
| PR2 | 0.9871 |
| RE1 | 0.9807 |
| RE3 | 0.988 |
| CL1 | 0.9791 |
| CL2 | 0.9412 |
| CL3 | 0.9384 |
| CO2 | 0.9887 |
| CO3 | 0.975 |
| IT1 | 0.9732 |
| QLX1 | 0.9825 |
| QL3 | 0.9741 |
| QL4 | 0.9631 |
| SSX1 | 0.9556 |
| SSX2 | 0.9579 |
| SCX1 | 0.9613 |
| SCX2 | 0.9583 |
| SW1 | 0.967 |
| SW2 | 0.9662 |
| SW3 | 0.969 |
| CWX3 | 0.9848 |
| CW4r | 0.987 |
| CW5 | 0.9866 |
| WE1 | 0.9826 |
| WE2 | 0.9259 |
| WE3 | 0.9031 |
| JI1 | 0.8608 |
| JI2 | 0.9053 |
| JI3 | 0.8768 |
| IW1 | 0.9226 |
| IW2 | 0.9715 |
| IW3 | 0.9517 |
| IW4 | 0.9528 |
| IW5 | 0.9698 |
| QW1 | 0.9874 |
| QW2 | 0.9837 |
| JS1 | 0.9735 |
| JS2 | 0.9883 |
| JS3 | 0.9854 |
| JS4 | 0.9831 |
| JS5 | 0.9713 |
| WF2 | 0.9616 |
| WF3 | 0.9458 |
| WF5 | 0.9661 |
| TE1r | 0.9177 |
| TE2r | 0.9264 |
| TE3 | 0.9704 |
| TM1 | 0.9791 |
| TMX2 | 0.9717 |
| TM3r | 0.965 |
| TM4 | 0.9838 |
| JU1 | 0.971 |
| JU2 | 0.9806 |
| JU4 | 0.9884 |
| Overall | 0.9652 |

S2 Table. Confirmatory factor analysis.

| Factor | Item | Estimate | SE | Z | p |
| --- | --- | --- | --- | --- | --- |
| QD | QD1 | 0.662 | 0.00838 | 79.0 | < .001 |
|  | QD2 | 0.780 | 0.00803 | 97.1 | < .001 |
|  | QD3 | 0.812 | 0.00803 | 101.1 | < .001 |
| WP | WP1 | 0.631 | 0.00703 | 89.8 | < .001 |
|  | WP2 | 0.739 | 0.00811 | 91.1 | < .001 |
| CD | CD1 | 0.713 | 0.00929 | 76.7 | < .001 |
|  | CD2 | 0.602 | 0.00725 | 83.1 | < .001 |
|  | CD3 | 0.271 | 0.00895 | 30.3 | < .001 |
|  | CD4 | 0.407 | 0.00909 | 44.8 | < .001 |
| ED | ED1 | 0.603 | 0.00688 | 87.6 | < .001 |
|  | EDX2 | 0.586 | 0.00964 | 60.8 | < .001 |
|  | ED3 | 0.798 | 0.00839 | 95.1 | < .001 |
| HE | HE1 | 0.536 | 0.01462 | 36.6 | < .001 |
|  | HE2 | 0.685 | 0.00997 | 68.7 | < .001 |
|  | HE3 | 0.458 | 0.00980 | 46.8 | < .001 |
|  | HE4 | 0.527 | 0.00995 | 53.0 | < .001 |
| IN | INX1 | 0.616 | 0.00835 | 73.8 | < .001 |
|  | IN2 | 0.511 | 0.01106 | 46.2 | < .001 |
|  | IN3 | 0.570 | 0.00881 | 64.7 | < .001 |
|  | IN4 | 0.709 | 0.00833 | 85.1 | < .001 |
| PD | PD2 | 0.649 | 0.00858 | 75.6 | < .001 |
|  | PD3 | 0.400 | 0.00743 | 53.8 | < .001 |
|  | PD4 | 0.755 | 0.00863 | 87.5 | < .001 |
| VA | VA1 | 0.542 | 0.01752 | 30.9 | < .001 |
|  | VA2 | -0.242 | 0.01046 | -23.2 | < .001 |
| CT | CT1 | 0.780 | 0.01102 | 70.8 | < .001 |
|  | CT2 | 0.574 | 0.01183 | 48.5 | < .001 |
|  | CT3 | 0.683 | 0.00976 | 69.9 | < .001 |
|  | CT4 | 0.820 | 0.01340 | 61.2 | < .001 |
|  | CT5 | -0.249 | 0.01044 | -23.9 | < .001 |
| MW | MW1 | 0.575 | 0.00690 | 83.3 | < .001 |
|  | MW2 | 0.475 | 0.00677 | 70.2 | < .001 |
| PR | PR1 | 0.656 | 0.00919 | 71.3 | < .001 |
|  | PR2 | 0.587 | 0.00713 | 82.3 | < .001 |
| RE | RE1 | 0.812 | 0.00903 | 90.0 | < .001 |
|  | RE3 | 0.707 | 0.00774 | 91.4 | < .001 |
| CL | CL1 | 0.505 | 0.00747 | 67.7 | < .001 |
|  | CL2 | 0.537 | 0.00679 | 79.1 | < .001 |
|  | CL3 | 0.530 | 0.00654 | 81.0 | < .001 |
| CO | CO2 | 0.610 | 0.00951 | 64.2 | < .001 |
|  | CO3 | 0.542 | 0.00848 | 63.9 | < .001 |
| IT | IT1 | 0.948 | 0.00683 | 138.7 | < .001 |
| QL | QLX1 | 0.811 | 0.00842 | 96.4 | < .001 |
|  | QL3 | 0.760 | 0.00842 | 90.3 | < .001 |
|  | QL4 | 0.824 | 0.00928 | 88.7 | < .001 |
| SS | SSX1 | 0.938 | 0.00850 | 110.4 | < .001 |
|  | SSx2 | 0.965 | 0.00846 | 114.0 | < .001 |
| SC | SCX1 | 0.554 | 0.00628 | 88.2 | < .001 |
|  | SCX2 | 0.598 | 0.00680 | 87.9 | < .001 |
| SW | SW1 | 0.450 | 0.00553 | 81.3 | < .001 |
|  | SW2 | 0.470 | 0.00560 | 84.0 | < .001 |
|  | SW3 | 0.650 | 0.00709 | 91.6 | < .001 |
| CW | CWX3 | 0.880 | 0.00878 | 100.3 | < .001 |
|  | CW4 | -0.834 | 0.01093 | -76.3 | < .001 |
|  | CW5 | 0.737 | 0.00816 | 90.3 | < .001 |
| WE | WE1 | 0.447 | 0.00869 | 51.4 | < .001 |
|  | WE2 | 0.590 | 0.00754 | 78.3 | < .001 |
|  | WE3 | 0.555 | 0.00875 | 63.4 | < .001 |
| JI | JI1 | 0.446 | 0.00734 | 60.8 | < .001 |
|  | JI2 | 0.383 | 0.00716 | 53.5 | < .001 |
|  | JI3 | 0.630 | 0.01044 | 60.4 | < .001 |
| IW | IW1 | 0.729 | 0.01050 | 69.5 | < .001 |
|  | IW2 | 0.787 | 0.01052 | 74.8 | < .001 |
|  | IW3 | 0.860 | 0.01118 | 76.9 | < .001 |
|  | IW4 | 0.787 | 0.01248 | 63.1 | < .001 |
|  | IW5 | -0.369 | 0.01138 | -32.4 | < .001 |
| QW | QW1 | 0.523 | 0.00716 | 73.0 | < .001 |
|  | QW2 | 0.495 | 0.00749 | 66.1 | < .001 |
| JS | JS1 | 0.647 | 0.00806 | 80.2 | < .001 |
|  | JS2 | 0.552 | 0.01031 | 53.5 | < .001 |
|  | JS3 | 0.614 | 0.00784 | 78.3 | < .001 |
|  | JS4 | 0.654 | 0.00684 | 95.6 | < .001 |
|  | JS5 | 0.386 | 0.01088 | 35.5 | < .001 |
| WF | WF2 | 0.980 | 0.00938 | 104.4 | < .001 |
|  | WF3 | 0.969 | 0.00927 | 104.5 | < .001 |
|  | WF5 | 0.930 | 0.00917 | 101.3 | < .001 |
| TE | TE1 | 0.538 | 0.01125 | 47.9 | < .001 |
|  | TE2 | 0.535 | 0.01219 | 43.9 | < .001 |
|  | TE3 | -0.420 | 0.00972 | -43.2 | < .001 |
| TM | TM1 | 0.477 | 0.00711 | 67.1 | < .001 |
|  | TM2 | 0.647 | 0.00744 | 86.9 | < .001 |
|  | TM4 | 0.621 | 0.00799 | 77.8 | < .001 |
|  | TM3 | -0.588 | 0.00875 | -67.2 | < .001 |
| JU | JU1 | 0.697 | 0.00779 | 89.5 | < .001 |
|  | JU2 | 0.790 | 0.00922 | 85.7 | < .001 |
|  | JU4 | 0.470 | 0.00762 | 61.6 | < .001 |

| χ^2^ | df | p |  |  |
| --- | --- | --- | --- | --- |
| 52330 | 3164 | < .001 |  |  |
| CFI | TLI |  |  |  |
| 0.88 | 0.862 |  |  |  |
|  |  | RMSEA 90% CI | |  |
|  |  | lower | upper |  |
| RMSEA | 0.0397 | 0.0394 | 0.04 |  |
|  |  |  |  |  |
|  | mean | sd | Cronbach’s α | McDonald’s ω |
| Scale Reliability Statistics | 2.96 | 0.813 | 0.847 | 0.85 |

S3 Table. Exploratory factor analysis, maximum likelihood and factor loadings.

| Variable | Factor1 | Factor2 | Factor3 | Factor4 | Factor5 | Factor6 | Factor7 | Factor8 | Factor9 | Factor10 | Factor11 | Factor12 | Factor13 | Factor14 | Factor15 |
| --- | --- | --- | --- | --- | --- | --- | --- | --- | --- | --- | --- | --- | --- | --- | --- |
| QD1 | -0.1444 | -0.0652 | 0.2106 | 0.6333 | 0.0344 | 0.1229 | -0.0854 | 0.0957 | 0.0172 | -0.0148 | 0.0311 | 0.0144 | -0.0341 | -0.0224 | -0.0021 |
| QD2 | -0.0986 | -0.0521 | 0.2178 | 0.7744 | 0.04 | 0.1401 | -0.0595 | 0.1087 | -0.0116 | 0.0177 | 0.0326 | -0.012 | -0.0325 | -0.0189 | -0.0078 |
| QD3 | -0.0811 | -0.0337 | 0.2387 | 0.8 | 0.02 | 0.1409 | -0.0923 | 0.096 | 0.0156 | 0.0324 | 0.0293 | -0.0068 | -0.0293 | -0.0142 | 0.0034 |
| WP1 | -0.1157 | -0.0118 | 0.2193 | 0.3004 | 0.0519 | 0.1135 | 0.046 | 0.3417 | -0.1895 | -0.1316 | 0.0642 | 0.0132 | -0.0356 | -0.0301 | -0.0049 |
| WP2 | -0.0697 | 0.0065 | 0.2259 | 0.3398 | 0.0387 | 0.0835 | 0.1106 | 0.3011 | -0.1765 | -0.0699 | 0.1128 | 0.0333 | -0.0244 | -0.0124 | 0.0347 |
| CD1 | -0.0686 | 0.0047 | 0.1806 | 0.1234 | 0.061 | 0.0936 | 0.0379 | 0.7344 | -0.1836 | -0.0846 | 0.034 | 0.0273 | -0.0205 | -0.0274 | 0.0218 |
| CD2 | -0.0284 | 0.0154 | 0.189 | 0.2113 | 0.0321 | 0.1099 | 0.0412 | 0.7113 | -0.0471 | -0.0509 | 0.0803 | 0.0498 | -0.0171 | -0.0131 | 0.0664 |
| CD3 | 0.0436 | -0.0148 | 0.0642 | 0.1289 | 0.0197 | 0.1007 | -0.0287 | 0.254 | 0.1495 | 0.2457 | 0.1983 | 0.0804 | 0.0184 | -0.0374 | 0.0263 |
| CD4 | -0.0838 | -0.0399 | 0.1529 | 0.218 | 0.0257 | 0.348 | -0.0095 | 0.2876 | 0.033 | 0.0955 | 0.0808 | 0.0246 | 0.0191 | -0.0857 | 0.0057 |
| ED1 | -0.0984 | -0.0177 | 0.1818 | 0.1661 | 0.0431 | 0.7272 | -0.0268 | 0.115 | -0.0562 | -0.0097 | 0.0397 | 0.0363 | -0.0138 | -0.0283 | 0.0207 |
| EDX2 | -0.0838 | 0.0094 | 0.1141 | 0.1764 | 0.0249 | 0.551 | -0.0734 | 0.0029 | 0.0437 | 0.0451 | 0.0128 | -0.0014 | -0.0377 | -0.0125 | -0.0297 |
| ED3 | -0.1126 | -0.0169 | 0.2129 | 0.1434 | 0.0636 | 0.8263 | -0.0057 | 0.061 | -0.0474 | -0.025 | 0.0363 | 0.0067 | -0.0353 | -0.0349 | 0.058 |
| HE1 | -0.0411 | -0.017 | 0.0911 | 0.0394 | 0.056 | 0.0562 | 0.0353 | 0.1882 | -0.0177 | -0.0479 | 0.042 | -0.0158 | -0.0196 | -0.0392 | -0.0297 |
| HE2 | -0.1743 | -0.0903 | 0.2294 | 0.0777 | 0.0806 | 0.307 | -0.0221 | 0.1619 | -0.0771 | -0.0757 | -0.0554 | -0.0021 | -0.078 | -0.062 | -0.0316 |
| HE3 | -0.0665 | -0.0159 | 0.1337 | 0.0829 | 0.0584 | 0.1805 | 0.012 | 0.1724 | -0.0449 | -0.0784 | 0.032 | -0.0203 | -0.0238 | 0.0156 | 0.0755 |
| HE4 | -0.2123 | -0.098 | 0.1206 | 0.0542 | 0.0843 | 0.1201 | -0.0545 | 0.1104 | -0.0816 | -0.091 | -0.0356 | -0.0411 | -0.0522 | -0.0731 | -0.0506 |
| INX1 | 0.3268 | 0.0634 | -0.0877 | 0.0275 | -0.093 | 0.0094 | 0.0717 | -0.0493 | 0.1572 | 0.5475 | 0.0962 | 0.1397 | 0.0877 | 0.02 | 0.0287 |
| IN2 | 0.1621 | 0.1321 | -0.064 | 0.0447 | -0.0535 | -0.0268 | -0.0046 | -0.0924 | 0.2606 | 0.3465 | 0.0406 | 0.0474 | 0.0761 | -0.0702 | -0.0166 |
| IN3 | 0.2027 | -0.0056 | -0.1275 | -0.0527 | -0.0198 | -0.0068 | -0.0359 | -0.1156 | 0.2715 | 0.5129 | 0.057 | 0.0755 | 0.0473 | -0.0201 | -0.0132 |
| IN4 | 0.2231 | 0.0471 | -0.0974 | 0.0257 | -0.0569 | -0.0183 | 0.0094 | -0.0622 | 0.2259 | 0.7461 | 0.0878 | 0.1003 | 0.0652 | -0.0098 | 0.0353 |
| PD2 | 0.3191 | 0.1003 | -0.0409 | 0.01 | -0.0403 | 0.0244 | 0.0166 | 0.0767 | 0.056 | 0.1082 | 0.1111 | 0.6718 | 0.1237 | 0.039 | 0.1301 |
| PD3 | 0.1486 | 0.1467 | -0.0263 | 0.009 | -0.0422 | 0.1334 | 0.2641 | 0.1075 | -0.0187 | 0.0438 | 0.16 | 0.3259 | 0.1027 | 0.0389 | 0.3363 |
| PD4 | 0.3973 | 0.1138 | -0.0823 | -0.0357 | -0.0694 | 0.0176 | 0.1647 | 0.0137 | 0.0727 | 0.1475 | 0.1146 | 0.6277 | 0.1512 | 0.0362 | 0.0869 |
| VA1 | 0.103 | 0.1221 | -0.0222 | 0.1402 | -0.0393 | 0.1214 | 0.0396 | 0.1501 | -0.0293 | 0.1396 | 0.1502 | 0.3109 | 0.0772 | 0.0049 | 0.1995 |
| VA2r | 0.0738 | 0.0091 | -0.0965 | -0.001 | -0.0686 | 0.0098 | -0.0826 | -0.1607 | 0.0619 | 0.1691 | 0.0796 | 0.2203 | 0.0504 | 0.0515 | 0.081 |
| CT1 | 0.1617 | 0.0339 | -0.1366 | -0.0804 | -0.0827 | -0.0688 | -0.0133 | -0.092 | 0.6141 | 0.2317 | 0.0245 | 0.037 | 0.0354 | 0.001 | -0.0139 |
| CT2 | 0.1958 | 0.0021 | -0.1172 | -0.04 | -0.0924 | -0.0479 | 0.0216 | -0.098 | 0.4211 | 0.17 | 0.0496 | 0.0502 | 0.0519 | 0.0367 | 0.0012 |
| CT3 | 0.1525 | 0.0659 | -0.1065 | -0.0243 | -0.0731 | -0.0608 | -0.0318 | -0.1542 | 0.6058 | 0.2125 | 0.022 | 0.0276 | 0.0511 | 0.0049 | -0.0232 |
| CT4 | 0.1037 | -0.0236 | -0.0603 | 0.1089 | -0.1299 | -0.0274 | -0.0473 | -0.1461 | 0.5858 | 0.2615 | 0.0407 | 0.0779 | 0.0186 | 0.033 | -0.0088 |
| CT5r | 0.087 | 0.0262 | -0.2664 | -0.3139 | -0.0139 | -0.0608 | 0.0347 | -0.2136 | 0.1164 | 0.025 | -0.0769 | -0.0773 | 0.0324 | 0.0305 | -0.0353 |
| MW1 | 0.2089 | 0.1638 | -0.1328 | -0.0126 | -0.0523 | 0.0762 | 0.2057 | 0.0305 | -0.0229 | 0.0546 | 0.2731 | 0.2118 | 0.1319 | 0.0534 | 0.6129 |
| MW2 | 0.1687 | 0.1581 | -0.0681 | -0.0001 | -0.0391 | 0.0758 | 0.1867 | 0.1341 | -0.0314 | -0.0069 | 0.3143 | 0.1076 | 0.1157 | 0.0441 | 0.5452 |
| PR1 | 0.5702 | 0.0556 | -0.0971 | -0.0561 | -0.1372 | -0.047 | 0.1454 | -0.0583 | 0.0602 | 0.1466 | 0.0294 | 0.1107 | 0.0807 | 0.0701 | -0.0012 |
| PR2 | 0.5598 | 0.1807 | -0.124 | -0.1432 | -0.0702 | -0.0615 | 0.276 | -0.0322 | 0.0268 | 0.0546 | 0.0527 | 0.1172 | 0.0749 | 0.0914 | 0.0562 |
| RE1 | 0.6872 | 0.0941 | -0.1259 | -0.059 | -0.0962 | -0.0587 | 0.1161 | -0.0282 | 0.0511 | 0.1107 | 0.0874 | 0.1113 | 0.1047 | 0.0318 | 0.0687 |
| RE3 | 0.6482 | 0.2509 | -0.1403 | -0.0712 | -0.1031 | -0.0618 | 0.0974 | -0.0288 | 0.0341 | 0.0695 | 0.0396 | 0.0829 | 0.125 | 0.0764 | 0.0522 |
| CL1 | 0.3047 | 0.1246 | -0.0672 | -0.1259 | -0.0143 | -0.0628 | 0.4878 | 0.0729 | -0.0372 | 0.0095 | 0.0787 | 0.1345 | 0.0884 | 0.0765 | 0.1142 |
| CL2 | 0.1903 | 0.1318 | -0.0879 | -0.1161 | -0.0746 | -0.0326 | 0.7117 | 0.0078 | -0.007 | 0.0181 | 0.0855 | 0.0372 | 0.0666 | 0.0414 | 0.1227 |
| CL3 | 0.2327 | 0.1607 | -0.0738 | -0.1018 | -0.0357 | -0.0203 | 0.7269 | 0.0584 | -0.0353 | -0.0054 | 0.0653 | 0.0541 | 0.0202 | 0.0606 | 0.0606 |
| CO2 | -0.3194 | -0.1171 | 0.2065 | 0.3086 | 0.0874 | 0.1865 | -0.2179 | 0.1005 | -0.0045 | -0.0085 | 0.0205 | -0.0436 | -0.0455 | -0.1032 | -0.0403 |
| CO3 | -0.2252 | -0.0661 | 0.2147 | 0.2618 | 0.0759 | 0.1698 | -0.1271 | 0.1284 | -0.0558 | -0.014 | -0.0394 | -0.0085 | -0.0739 | -0.0985 | -0.035 |
| IT1 | -0.2244 | -0.0298 | 0.2238 | 0.231 | 0.1342 | 0.1479 | -0.0749 | 0.1327 | -0.0364 | -0.0443 | -0.0874 | -0.0703 | -0.0923 | -0.0724 | -0.0867 |
| QLX1 | 0.7451 | 0.0988 | -0.0968 | -0.0637 | -0.0366 | -0.0284 | 0.0473 | -0.0446 | 0.0778 | 0.0859 | 0.0403 | 0.2536 | 0.1035 | 0.0293 | 0.0136 |
| QL3 | 0.747 | 0.0798 | -0.0767 | -0.0792 | -0.0123 | -0.0483 | 0.0838 | -0.0298 | 0.0057 | 0.0267 | 0.0453 | 0.0543 | 0.0224 | 0.0325 | 0.0353 |
| QL4 | 0.8044 | 0.0893 | -0.0581 | -0.0055 | -0.0255 | -0.0308 | 0.0166 | -0.0258 | 0.017 | 0.025 | 0.0238 | -0.0015 | 0.0009 | 0.0584 | 0.0108 |
| SSX1 | 0.8067 | 0.183 | -0.0718 | -0.0517 | -0.046 | -0.0403 | 0.0412 | -0.0007 | 0.0076 | 0.0349 | 0.0337 | -0.0035 | -0.0019 | 0.043 | 0.0375 |
| SSX2 | 0.811 | 0.1953 | -0.0819 | -0.0454 | -0.0316 | -0.0527 | 0.0471 | 0.0011 | 0.043 | 0.0511 | 0.0439 | -0.0071 | 0.0337 | 0.0267 | 0.0219 |
| SCX1 | 0.255 | 0.7289 | -0.0848 | -0.0778 | -0.0066 | -0.0142 | 0.0565 | 0.0342 | 0.0156 | 0.0143 | 0.0219 | 0.0586 | 0.0531 | 0.0471 | 0.0378 |
| SCX2 | 0.2436 | 0.734 | -0.0585 | -0.0401 | -0.0331 | 0.0144 | 0.0685 | -0.0076 | 0.0022 | 0.0217 | 0.0161 | 0.0567 | 0.0298 | 0.0781 | 0.059 |
| SW1 | 0.2459 | 0.6907 | -0.0809 | -0.0242 | -0.0174 | -0.0308 | 0.0724 | -0.0161 | 0.0414 | 0.023 | 0.0961 | 0.0116 | 0.0189 | 0.106 | 0.0263 |
| SW2 | 0.2701 | 0.6931 | -0.0938 | -0.0308 | -0.007 | -0.013 | 0.0756 | -0.0041 | 0.0212 | 0.0125 | 0.0661 | 0.0356 | 0.0449 | 0.095 | 0.0228 |
| SW3 | 0.2792 | 0.7281 | -0.0824 | -0.0104 | -0.0572 | -0.0205 | 0.0969 | 0.0029 | -0.0074 | 0.0397 | 0.0883 | 0.0433 | 0.0915 | 0.0604 | 0.0647 |
| CWX3 | 0.5792 | 0.2033 | -0.2193 | -0.1327 | -0.0886 | -0.0648 | 0.0808 | -0.0196 | 0.0714 | 0.1106 | 0.1095 | 0.1437 | 0.2413 | 0.057 | 0.1381 |
| CW4r | 0.3608 | 0.1277 | -0.3464 | -0.2112 | -0.1506 | -0.1401 | 0.0758 | -0.0743 | 0.0313 | 0.0733 | 0.1397 | 0.0898 | 0.2906 | 0.0399 | 0.1294 |
| CW5 | 0.5107 | 0.194 | -0.1602 | -0.0978 | -0.1252 | -0.0577 | 0.1151 | -0.0101 | 0.0479 | 0.0866 | 0.1782 | 0.1388 | 0.255 | 0.0435 | 0.1987 |
| WE1 | 0.2505 | 0.1615 | -0.3319 | -0.127 | -0.0843 | -0.0909 | 0.097 | -0.0179 | 0.0582 | 0.0922 | 0.4217 | 0.103 | 0.1398 | 0.0057 | 0.0951 |
| WE2 | 0.1639 | 0.1415 | -0.0561 | 0.041 | -0.0434 | 0.0522 | 0.1052 | 0.0553 | 0.0296 | 0.0696 | 0.7657 | 0.072 | 0.0777 | 0.0269 | 0.1504 |
| WE3 | 0.1367 | 0.0464 | 0.0425 | 0.1147 | -0.0152 | 0.066 | 0.0252 | 0.0814 | 0.0257 | 0.0837 | 0.6583 | 0.0999 | 0.0811 | 0.0142 | 0.0878 |
| JI1 | -0.0454 | -0.1003 | 0.0233 | -0.0246 | 0.2623 | 0.0285 | -0.0481 | -0.0244 | -0.0035 | -0.0136 | 0.0215 | 0.0139 | -0.0887 | -0.0438 | -0.0219 |
| JI2 | -0.0394 | -0.0712 | 0.0288 | 0.0028 | 0.3512 | -0.0107 | -0.0391 | -0.0273 | -0.0276 | 0.0402 | -0.0184 | -0.0196 | 0.0342 | -0.0886 | -0.0686 |
| JI3 | -0.0817 | -0.0823 | 0.0415 | 0.0031 | 0.3049 | 0.0165 | -0.0118 | -0.0473 | 0.032 | -0.0249 | -0.0519 | -0.0576 | -0.0503 | -0.0235 | -0.0373 |
| IW1 | -0.151 | -0.0456 | 0.0732 | 0.0365 | 0.7052 | 0.0248 | -0.037 | -0.0015 | 0.0162 | -0.023 | -0.0295 | -0.0328 | -0.0376 | -0.0417 | -0.0101 |
| IW2 | -0.2507 | -0.0612 | 0.1806 | 0.1139 | 0.5802 | 0.098 | -0.0809 | 0.0493 | -0.0076 | -0.045 | -0.0269 | -0.0529 | -0.1309 | -0.0594 | -0.0203 |
| IW3 | -0.2203 | -0.0225 | 0.1585 | 0.0266 | 0.6942 | 0.0725 | -0.0407 | 0.0772 | -0.1736 | -0.0814 | -0.0368 | -0.041 | -0.0657 | -0.0589 | -0.0202 |
| IW4 | -0.1272 | -0.0177 | 0.1866 | 0.0275 | 0.5403 | 0.0614 | -0.0233 | 0.0708 | -0.1492 | -0.0445 | -0.037 | -0.0138 | -0.0623 | -0.0627 | -0.0508 |
| IW5 | 0.2949 | 0.1009 | -0.1143 | -0.0335 | -0.0939 | -0.0341 | 0.0773 | -0.0088 | 0.0418 | 0.0718 | 0.0798 | 0.1459 | 0.5257 | 0.0241 | 0.0932 |
| QW1 | 0.3652 | 0.1473 | -0.2293 | -0.3398 | -0.0577 | -0.1029 | 0.2021 | -0.0453 | 0.101 | 0.0815 | 0.1164 | 0.1051 | 0.1261 | 0.0345 | 0.0905 |
| QW2 | 0.3957 | 0.2504 | -0.1357 | -0.1303 | -0.0556 | -0.0782 | 0.1361 | 0.0024 | 0.0767 | 0.0337 | 0.0877 | 0.1036 | 0.1338 | 0.1312 | 0.0917 |
| JS1 | 0.3922 | 0.1379 | -0.1832 | -0.0726 | -0.1224 | -0.0522 | 0.0707 | -0.0344 | 0.0491 | 0.0954 | 0.1399 | 0.1578 | 0.6831 | 0.0223 | 0.0856 |
| JS2 | 0.2739 | 0.0788 | -0.1989 | -0.1213 | -0.0898 | -0.0934 | 0.0323 | -0.09 | 0.1947 | 0.122 | 0.1188 | 0.0904 | 0.2058 | 0.0368 | 0.0648 |
| JS3 | 0.4575 | 0.1761 | -0.1393 | -0.0534 | -0.1137 | -0.0334 | 0.1218 | -0.0071 | 0.0558 | 0.0689 | 0.1053 | 0.2658 | 0.2558 | 0.0731 | 0.1153 |
| JS4 | 0.4367 | 0.205 | -0.2528 | -0.1228 | -0.09 | -0.0682 | 0.0972 | -0.0141 | 0.071 | 0.0718 | 0.214 | 0.1366 | 0.3727 | 0.0566 | 0.1613 |
| JS5 | 0.1936 | 0.0083 | -0.1774 | -0.0799 | -0.1336 | -0.1204 | -0.0306 | -0.104 | 0.1204 | 0.0673 | 0.076 | 0.082 | 0.1568 | -0.0009 | -0.0005 |
| WF2 | -0.1987 | -0.0994 | 0.76 | 0.2173 | 0.0961 | 0.1883 | -0.0373 | 0.1205 | -0.0767 | -0.086 | -0.047 | -0.0563 | -0.0915 | -0.0299 | -0.0321 |
| WF3 | -0.1656 | -0.087 | 0.8305 | 0.1699 | 0.0882 | 0.1158 | -0.0426 | 0.1069 | -0.0716 | -0.0421 | -0.0067 | -0.0296 | -0.0613 | -0.0545 | -0.0385 |
| WF5 | -0.2169 | -0.1074 | 0.7444 | 0.2441 | 0.1079 | 0.1596 | -0.0816 | 0.0985 | -0.0215 | -0.0395 | -0.0194 | -0.0051 | -0.0458 | -0.0506 | -0.0235 |
| TE1r | 0.1878 | 0.2923 | -0.0524 | -0.0243 | -0.0553 | -0.0285 | 0.0564 | -0.0093 | -0.01 | -0.026 | 0.0185 | 0.0367 | 0.005 | 0.6921 | 0.0221 |
| TE2r | 0.269 | 0.1305 | -0.084 | -0.0363 | -0.0557 | -0.0487 | 0.0665 | -0.0325 | 0.0136 | 0.0004 | 0.0273 | 0.0187 | 0.0281 | 0.7431 | 0.0361 |
| TE3 | 0.2973 | 0.4704 | -0.0548 | -0.034 | -0.0274 | -0.0053 | 0.088 | -0.013 | -0.0016 | 0.0036 | 0.0652 | 0.0737 | 0.0343 | 0.1895 | 0.0532 |
| TM1 | 0.4801 | 0.1668 | -0.0514 | -0.054 | -0.0751 | -0.0149 | 0.1292 | 0.0203 | 0.0246 | 0.023 | 0.0571 | 0.0433 | 0.0542 | 0.1206 | 0.0592 |
| TMX2 | 0.6344 | 0.0743 | -0.0672 | -0.0197 | -0.1295 | -0.0366 | 0.0679 | -0.0116 | 0.0523 | 0.0412 | 0.0564 | 0.0361 | 0.0581 | 0.1465 | 0.0155 |
| TM3r | 0.4794 | 0.0194 | -0.0783 | -0.0295 | -0.1521 | -0.0638 | 0.027 | -0.031 | 0.0641 | 0.0216 | 0.0081 | 0.0456 | 0.0396 | 0.4724 | 0.0056 |
| TM4 | 0.5796 | 0.2379 | -0.0542 | -0.0233 | -0.1037 | -0.0129 | 0.0255 | -0.0084 | 0.0707 | 0.0973 | 0.045 | 0.0476 | 0.0608 | 0.1411 | -0.0046 |
| JU1 | 0.7349 | 0.1383 | -0.0593 | -0.0108 | -0.086 | -0.0391 | 0.028 | -0.0209 | 0.0567 | 0.0417 | 0.0168 | 0.0221 | 0.034 | 0.1135 | -0.012 |
| JU2 | 0.6848 | 0.0895 | -0.1244 | -0.0472 | -0.0695 | -0.0452 | 0.0248 | -0.0585 | 0.1064 | 0.0948 | 0.0555 | 0.055 | 0.0682 | 0.0637 | 0.0189 |
| JU4 | 0.4356 | 0.2362 | -0.1611 | -0.1517 | -0.114 | -0.0519 | 0.0936 | -0.0378 | 0.0626 | 0.0597 | 0.0389 | 0.0697 | 0.102 | 0.1228 | 0.0517 |
