# Supplementary material for: The Norwegian version of the Copenhagen Psychosocial Questionnaire (COPSOQ III): Initial validation study using a national sample of registered nurses
Source: PLoS One. 2023 Aug 24;18(8):e0289739. doi: 10.1371/journal.pone.0289739 (PMC10449149; doi:10.1371/journal.pone.0289739)
Supplement: Attachment — Submitted filename: Response to reviewers10April2023.docx [file pone.0289739.s003.docx]

Response to reviewers

Reviewers' comments:
Reviewer #1: Review of PONE-D-22-27893 – The Norwegian version of the Copenhagen Psychosocial Questionnaire (COPSOQ III): Initial validation study using a national sample of licensed nurses

Main justification for the recommended decision

The authors report the results of a validation study of the Copenhagen Psychosocial Questionnaire (COPSOQ III) translated into Norwegian. I enjoyed reading the manuscript, and I think the authors provide a well-informed justification for the focus on the psychosocial work environment, for why there is a need for more translated versions of the COPSOQ, and for the methods they used to assess the translated measurement instrument. The translation and the analyses appear very sound. I commend the authors for their contribution to the literature by improving the chances for building knowledge about the psychosocial work environment across countries. I also think it is good that the authors not only used item response theory models but also factor analyses for triangulation and increased comprehension.

Response: Thank you for your careful reading and reviewing of the manuscript! Your comments are much appreciated, and we have tried to respond to all of them and we have revised the manuscript accordingly.

I recommend that the manuscript goes through a minor revision prior to resubmission. My main concern is the content of the introduction, and the general structure of the manuscript. I address each of these in turn, and I also include a list of more specific comments at the end of the introduction.

Revising the introduction

In the introduction, there is no clear, conceptual definition of a psychosocial work environment. Nor is there any detail in the theories about the psychosocial work environment that the authors mention. Finally, there is no description of other scales used for assessing the psychosocial work environment, or for why the COPSOQ is superior.
I think the authors should address the psychosocial work environment as a concept in much more detail in their introduction. What is a psychosocial work environment, i.e., what conceptual dimensions do we assume it consists of?

Response: Yes, thank you. Our objective was not to compare COPSOQ with other questionnaires that assess the psychosocial work environment. However, COPSOQ is by far the most used instrument and one of the reasons might be that an international network of researchers is established. See <https://www.copsoq-network.org/> if you are interested. We considered to include this web-page link in the manuscript, but decided not to - as it is easily found when googling COPSOQ.

However, we agree that the conceptual background and development of "psychosocial work environment" should be included, and we have added two new paragraphs to the introduction.

First, at the end of the subsection "Comparative research and validation of instruments across cultures" we have added:

An example of such an instrument is the Copenhagen Psychosocial Questionnaire (COPSOQ), which today is used worldwide in research and for workplace assessments [33].

33.	Burr, H., et al., The third version of the Copenhagen Psychosocial Questionnaire. Safety and health at work, 2019. 10(4): p. 482-503.

And under the heading "Psychosocial and organisational risk factors neglected in many OHS management system" we have added:

The concept of psychosocial work environment has gradually been developed with inspiration from the workplace accident prevention literature that in the 1950s used descriptions as "a social-psychological approach" [34], "psychological work environment" and "psychological climate of the workplace" [35]. In the 1960-70s the industrial democracy research introduced "psychological and social criteria for the evaluation of work situations" and "psychosocial aspects of the work environment" [36, 37], while the work stress research at the end of 1970s used the term "psychosocial effects of work environments" [38] and "psychosocial environment" [39].

In the early days, the main emphasis was on “psychosocial hazards”, which Coz and Griffiths defined as aspects of the "design and management of work and its social and organisational contexts that have the potential for causing psychological or physical harm" [40]. Over the years, it has become clear that a strict focus on avoiding risks that may lead to health-related problems is problematic and not necessarily leading to the wanted effects [41]. Thus, Leka et al argue for a need of also addressing psychosocial factors that can promote wellbeing and performance through organisational learning and development [38]. In line with this, Rugulies more recently suggests that the psychosocial work environment is important for understanding how the "interrelation of societal structures, environmental exposures, and psychological and psycho-physiological processes affect health and illness" [42]. In line with this extended understanding of the research field, the term “psychosocial work environment” has been replaced with “organisational and social work environment” in the newest Swedish regulations; thus, signalling a clear shift towards more emphasis on the contextual work factors rather than individual factors.

34.	Castle, P.F.C., Accidents, Absence, and Withdrawal from the Work Situation. Human Relations, 1956. 9(2): p. 223-233.

35.	Kerr, W., Complementary theories of safety psychology. The Journal of Social Psychology, 1957. 45(1): p. 3-9.

36.	Emery, F.E., et al., Democracy at work: The report of the Norwegian industrial democracy program. Vol. 2. 1976: Springer.

37.	Thorsrud, E., Democracy at work and perspectives on the quality of working life in Scandinavia. International Studies of Management & Organization, 1978. 8(1/2): p. 59-81.

38.	Karasek Jr, R.A., Job demands, job decision latitude, and mental strain: Implications for job redesign. Administrative science quarterly, 1979: p. 285-308.

39.	Johansson, G., G. Aronsson, and B. Lindstrom, Social psychological and neuroendocrine stress reactions in highly mechanised work. Ergonomics, 1978. 21(8): p. 583-599.

40.	Cox, T., A. Griffiths, and R. Randall, A risk management approach to the prevention of work stress, in The handbook of work and health psychology. 2003.

41.	Leka, S., W. Van Wassenhove, and A. Jain, Is psychosocial risk prevention possible? Deconstructing common presumptions. Safety science, 2015. 71: p. 61-67.

42.	Rugulies, R., What is a psychosocial work environment? Scandinavian journal of work, environment & health, 2019. 45(1): p. 1-6.

Which theories are used to explain the effects of a psychosocial work environment? What are the psychological explanations of why a better psychosocial work environment reduces sickness absenteeism and increases motivation for work?

Response: Yes, thank you. We included more references to this part:

Part a) is the focus of this study and this part covers multiple dimensions of work environment factors based on well-researched psychosocial theories such as Demand–Control–Support model [38], Effort–Reward Imbalance model [45], Job Demand-Resources model [46] and Workplace Social Capital [47]. The 28 dimensions in part a) are analysed based on 86 out of 99 items.

38.	Karasek Jr, R.A., Job demands, job decision latitude, and mental strain: Implications for job redesign. Administrative science quarterly, 1979: p. 285-308.

45.	Siegrist, J., Adverse health effects of high-effort/low-reward conditions. Journal of occupational health psychology, 1996. 1(1): p. 27.

46.	Demerouti, E., et al., The job demands-resources model of burnout. Journal of Applied psychology, 2001. 86(3): p. 499.

47.	Berthelsen, H., et al., Construct validity of a global scale for workplace social capital based on COPSOQ III. PloS one, 2019. 14(8): p. e0221893.

I realize that this is not a theoretical article on the psychosocial work environment, but I still believe that some conceptualization and contextualization is necessary in order to give a better description of the context, and in order to situate the COPSOQ instrument relative to our understanding of the psychosocial work environment and relative to alternative scales.

Response: Yes, there are alternative instruments, for instance QPS-Nordic, however not nearly as much researched as COPSOQ. However, we do not think it is necessary to discuss other instruments because this is a validation study of COPSOQ.

I refer the authors to the following papers as a starting point. I also note that the psychosocial work environment specifically is not my main area of expertise, and that the authors may know of better papers to use as sources and as inspiration for revising their introduction section.

• Nieuwenhuijsen, K., Bruinvels, D., & Frings-Dresen, M. (2010). Psychosocial work environment and stress-related disorders, a systematic review. Occupational Medicine, 60(4), 277-286.
• Solovieva, S., Lallukka, T., Virtanen, M., & Viikari-Juntura, E. (2013). Psychosocial factors at work, long work hours, and obesity: a systematic review. Scandinavian Journal of Work, Environment & Health, 241-258.
• Vazquez, A. C. S., Pianezolla, M., & Hutz, C. S. (2018). Assessment of psychosocial factors at work: A systematic review. Estudos de Psicologia (Campinas), 35, 5-13.

Response: Thank you for your kind effort, however, these are outcome studies and not validation studies. We hope that the new information now included in the introduction gives a better explanation of the concept of psychosocial work environment

Revising the remainder of the manuscript

Regarding the remainder of the manuscript, I believe that the following sections should be majorly revised or moved to other parts of the manuscript:

• Motive for the translation (lines 333-347) section in the discussion. Parts of this section can either be moved to the introduction or merged with the section on the empirical results. The main summary of the motive for the translation can be summarized much more succinctly.

Response: Yes, thank you - we agree and admit that there is not much discussion in the second paragraph. We have therefore moved this paragraph of the motivation section (lines 343-347) to the end of the Introduction section under the heading "This study", before the objective of the study, as it motivates the study.

• The Translation (lines 348-356) section in the discussion belongs in the description of the method. I would recommend moving this section to the Translation section of the Method section (lines 128-141).

Response: Yes, we agree. This text has been moved to the Translation section in Method.

• Other COPSOQ studies confirming measurement equivalence (lines 371-398) section in the discussion. This section has a detail level that I believe is unsuitable for the Discussion section, and it is quite unstructured. The authors should work to abstract the most important aspects of the other studies, and more clearly compare these studies, their contexts and their methods of analyses to their own.

Response: Yes, we see that this part is too detailed - so we have removed "‘Demands at work’, ‘Work organization and job contents’, ‘Interpersonal relations and leadership’, ‘Work-individual interface’, ‘Values at workplace level’, ‘Health and well-being’, and ‘Offensive behaviours’ and just included the references.

If this was an outcome study, we would do what you suggest. However, this is a validation study and adds to the COPSOQ literature rather than to be integrated through comparing the results as we do in outcome studies. COPSOQ III was published in 2019, so most of the published validation studies are based on previous versions, and thus not directly comparable to this version.

• Strengths and limitations (lines 405-409) section in the discussion. This is very brief, and not as informative as it could be. I would recommend that the authors go more into detail about the strengths and limitations of their translation process and their statistical analyses, as these are the most important aspects of their study that touch upon the contribution of the paper (a translated version of the COPSOQ with an initial validation study).

Response: Yes, thank you we have added the following text to the Strength and limitation section:

Another strength is that the translation was performed thorough a forward–backward translation process by a multidisciplinary team of researchers in collaboration with professional translators and an experienced linguist. The multidisciplinary team behind the translated version includes competence in labour economics, occupational medicine, physical rehabilitation medicine, organisational psychology, and COPSOQ expert competence.

• Further research (lines 410-416) section in the discussion. This section should be expanded following the addition of a new section to the introduction, as per the suggestions in this review. Additionally, it would be helpful with some more specific suggestions for future research also.
Response: Yes, thank you, this is a good suggestion. We have added the following:

The present study is the first validation study of the COPSOQ III instrument for use in the Norwegian context. However, validation is an ongoing process and future studies are needed for validation of the parts of the questionnaire not included here, using more diverse samples, investigation of other aspects of validity such as predictive validity and test-retest validity. Finally, we suggest that future research challenge the term "psychosocial work environment" by increased focus on contextual factors, which have a greater potential for designing workplace interventions that promote occupational health compared to individual factors.

More specific comments

• In the abstract
o Considering that the authors focus only on one of three parts of the COPSOQ in their validation study, I think this should be reflected in the abstract, specifically by adding a line about this in the Methods sections of the abstract (lines 31-38).

Response: Yes, thank you. We have now added the one sentence to the abstract Background and adjusted the next sentence so that it now reads:

COPSOQ III has three parts: a) work environment b) conflicts and offensive behaviours and c) health and welfare. We translated all three parts into Norwegian and evaluated the statistical properties of the 28 work environment dimensions in part a), using a sample of licensed nurses.

o The Conclusion section in the abstract should be elaborated on.

Response: Yes, the first paragraph of the conclusion now reads:

The established Norwegian translation of COPSOQ III can be used in further research about working environment factors and health and wellbeing in Norway. The extended use of the instrument internationally enables comparative studies, which can increase the knowledge and understanding of similarities and differences between labour markets in different countries.

The second paragraph now reads:

This first validation study shows that the Norwegian version has strong statistical properties like the original, and can be used to assess work environment factors, including relational and emotional risk factors and resources available at the workplace.

• Data availability
o The authors answer that the “Depersonalized data on the 86 items can be provided on request”. What is the justification for not uploading the data to a public repository? This should be included in the description.

Response: Yes, we changed this and have published the anonymous dataset at Norwegian Agency for Shared Services in Education and Research (Attribution-NonCommercial 4.0 International (CC BY-NC 4.0)). We still wait for a doi, and will provide this when we receive it.

• Introduction
o Lines 111-112: The phrasing of this sentence makes the meaning unclear. It may be worth rephrasing it to increase clarity.

Response: Yes, we have removed the last part of the sentence, because it is not very important to include this, and we have changed "Version III" to "The third version (COPSOQ III)" to make the sentence clearer.

o Lines 124-126: These theories are only mentioned by name and not elaborated on. This comment connects to the more general comment concerning a revision of the introduction, and should be seen in relation to that.

Response: Yes, thank you. We have included the original references behind the theories as explained above. These are all well known and well researched theories, so we do not think it is necessary to elaborate more on these. Hopefully, the added paragraph in the introduction introduces this field of research to the reader in a better way than before.

• Method
o Lines 148-149: What is the justification for using this cutoff?

Response: There is no standard for where to set the limit for inclusion in validation studies, however in statistical analyses in a validation study we do not want the results to be dependent of assumptions about missing values. That would add unnecessary complexity to the paper. How to treat missing values is a specific field of research that we did not want to bring into this study. Covering 85% (more than 112 of 133) allow us to not make a problem of missing data. We have now included a bit more information so that it now reads:

The first COPSOQ item was answered by 9844 nurses while 8784 answered the last question. In total, 8804 answered 85 % or more of the questions (more than 112 of all 132 items) and this is the sample used in this initial validation study.

Other researchers could make other choices. However, in a study where we wanted to test certain hypotheses, we would have robust-tested the results with different assumptions about missing values.

o Line 194: Typographical error. This should be “for dimensions that”.

Response: Thank you, we have corrected this.

o Line 207: Typographical error. Because the sentence refers to the items, and not the set, it should be “that together provide”.

Response: Thank you for this also, we have corrected this.

o Lines 209-212: Wordy sentence, please consider splitting the sentence into two and simplifying these for increased clarity.

Response: Yes, we have split the sentence in two to and it now reads:

More information (a higher value on the y-axis of the IIF) indicates more precise measurement at the continuum of θ. The value on the y-axis shows how much empirical information each item is adding to the dimension. The value on the x-axis shows where this empirical information is occurring along the continuum of the latent work environment variables (θ).

o Line 222: Which version of the jamovi software was used?

Response: Yes, thank you this is now included. Jamovi 2.2.5 was used.

• Kaiser-Meyer-Olkin measure (Lines 224-230).
o When describing this measure, the authors do not discuss the implication of their sample size for the suitability of the KMO values. Additionally, the reference that the authors refer to for suitability of different values does not support what they describe. This reference, which itself refers to a different reference that cannot be traced, for example refers to KMO values from 0.50 to 0.59 as “miserable”, not as “indicating the adequacy of the sample”. I would recommend that the authors refer to a more transparent source that also considers the role of sample size in determining suitability of KMO values, such as this one: http://pubs.sciepub.com/ajams/9/1/2

Response: Thank you for noticing this! The Beaver reference is commonly used; however, you are right – they do not refer to the original source which is Kaiser, 1974. We have now included the original reference, and it was Kaiser himself, and not Amy Beavers that stated the following: "in the .90s, marvelous in the .80s, meritorious in the .70s, middling in the .60s, mediocre in the .50s, miserable below .50, unacceptable".

As seen from S1 Table. Kaiser–Meyer–Olkin measure of sampling adequacy the lowest value in our sample is 0.88. This measure does not depend on sample size, and sample size is therefor not mentioned. Marvelous and meritorious imply that the data are very suitable for factor analysis.

• Method (continued)
o Line 232: The Norwegian word “og” should be replaced with the English “and”.
Response: Yes, thank you. This is now corrected.

• Results
o Line 243: Typographical error. Should be “Translation”

Response: Yes, thank you again. We have corrected this.

o Lines 268-270: The authors should go into more detail about how they deal with missing data.

Response: Yes, as explained above, to avoid the discussion on how to handle missing data, we have only included responses that have completed more than 85% of the questions.

• Discussion
o Lines 349-350: These claims are not substantiated by references and should be.

Response: There are no research or population studies that confirm this, language research is typically qualitative. There is however an international survey that shows that Norway and the Nordic countries generally speak English very well. We also learn English from the first grade in school. However, we decided to remove this sentence, as it is not important in this study. And the paragraph is now part of the Method section and not of the Discussion section as before, and should thus be tighten.

o Lines 354-356: These descriptions are fairly vague. It would help if the authors exemplified dialect words that were discussed or which specific choices they made in the translation process to keep an informal oral style.

Response: Yes, and this is meant to be general. Examples of Norwegian dialect words we discussed are in our view not of interest to the international reader. Dialect words were discussed especially when we discussed how we would formulate the questions in a natural and unformal or verbal way. One of the translators is "Østlending", one is "Trønder" and one is "Romsdaling", however this is not relevant to a non-Norwegian reader.

o Lines 408-409: This sentence is unfinished. There is one word, or several words, missing between “COPSOQ III” and “for the entire working population”

Response: Yes, thank you. We have now replaced the second sentence with the following: "The limitation is that the results are not representative for the entire working population, only for licenced nurses."

o Lines 418-420: This sentence should be divided in two for increased comprehensibility.

Response: Yes, thank you. We agree and the conclusion is rewritten as explained above.
Conclusion
Overall, I think this is a very promising manuscript, and I look forward to reading the revised version.

Response: Thank you for accepting to review our manuscript!

**********************************************************************************

Reviewer #2: The paper is very well written and covers an important area.
I have some (more general) comments.
1. And some points the authors talk about "the COPSOQ". They should make clear which of all possible items (CORE, middle, long) were used. All of them?

Response: Yes, thank you. This can be seen from "S1 Appendix. The translated items", see the fourth column.

2. Some of the items were identified as "candidate for removal". Are they from CORE, middle, or long? This information should be given in the text and could be added in table S1.

Response: Yes, thank you. We have now included the following sentence after Table 2:

Fourteen items are candidates to be removed (0 core items, 1 middle item and 13 long items), see table S1.

3. The study contains only one professional group. This is stated correctly at different points. However it should be made clearer that a lot of statistics (distribution of answers, i.e. dimension ED for a sample of nurses) and some findings maybe due to this fact. Expecially a decision on removal of items can never be based on such a sample (correct in line 294).

Response: Yes, we agree. And that is why we have included this. It should be clear from the text now that the results are based on a sample of licenced nurses.

4. Table 1: I would like to see some comments on bottom and ceiling effects.

Response: Yes, thank you. We have added the following text below Table 1:

The strongest ceiling effect is found in the Meaning of Work dimension (items MW1 and MW2) in this sample, and the strongest floor effect in the Job Insecurity (items JI1, JI2 and JI3), implying that the majority of nurses report that their work is meaningful and important, and that few nurses fear for their job.

5. Line 331: I would be interested in some more details what is congruent and what not compared to the English version but also to other validation studies.

Response: Yes, we think this would be interesting if we could find a study with a comparable sample. Unfortunately, we do not have a national sample that is representative for the working population in Norway as they have in Sweden and in Germany. We hope we will have that in the future or that other countries collect a national sample among licenced nurses.

We are aware of the German sample of nurses working in university hospitals (N = 567) and geriatric nurses in Germany (N=177), and the one from China covering hospital nurses (N=3088), one of oncology nurses (N = 63) in Brunei, hospital nurses in Estonia (N=404), female nurses at a university hospital in Seoul, South Korea (N=970) etc. so we believe that in the future it will be possible to do comparative analyses, and certainly when we start to do outcome analyses. However, we have added the following:

Using a corresponding approach, eight factors were identified in a broad sample from Germany [70], and the German standard version of COPSOQ III operates with five overarching domains as in previous versions [71].

70.	Kuczynski, I., et al., The assessment of psychosocial work conditions and their relationship to well-being: a multi-study report. International Journal of Environmental Research and Public Health, 2020. 17(5): p. 1654.

71.	Lincke, H.-J., et al., COPSOQ III in Germany: validation of a standard instrument to measure psychosocial factors at work. Journal of Occupational Medicine and Toxicology, 2021. 16: p. 1-15.

6. Line 371ff (up to 398): Here a (short!) comparison to the main stucture findings (factors, dimensions) of other validation studies would be very fruitful (France, Hungary, Germany, Chili, Turkey).

Response: Yes, we think this would be really fruitful if we could find comparable samples, i.e., a national sample of nurses from other validation studies. However, we do not think it will be wise to compare results from factor analyses of not-comparable samples as the results of statistical analyses on specific professions have strong sample dependency as you also pointed out above.

7. Explanation of IRT is lengthy (expecially the formulae from line 180-195).

Response: Yes, we can see that. However, we believe it is important to understand the method in order to understand the results, and IRT is unknown to many researchers who are not engaged in latent variable models. We suggest that this remains in the text, and those who are familiar with IRT can skip this part of the paper and those unfamiliar with the method can improve their understanding of IRT.

8. Table S2 and exp. S3. For a better orientation I would suggest to omit small values (in S3 i.e. < 0.3) or (better again) to mark higher values (i.e. > 0.4) in bold.

Response: Yes, thank you. We tried to make values higher than 0.4 in bold text, however, the readability of the table did not increase. Those very interested in these results could copy the table and paste it into Excel and perhaps use the Conditional Formatting tools available there. However, it is included as supplement data and not as part of the manuscript, as documentation more than results.

Comments to Editor from authors:

While revising your submission, please upload your figure files to the Preflight Analysis and Conversion Engine (PACE) digital diagnostic tool, [https://pacev2.apexcovantage.com/](https://eur03.safelinks.protection.outlook.com/?url=https%3A%2F%2Fpacev2.apexcovantage.com%2F&data=05%7C01%7Csolveig.ose%40sintef.no%7Cebe7732f4c5542db9d3a08db26740680%7Ce1f00f39604145b0b309e0210d8b32af%7C1%7C0%7C638146052159649758%7CUnknown%7CTWFpbGZsb3d8eyJWIjoiMC4wLjAwMDAiLCJQIjoiV2luMzIiLCJBTiI6Ik1haWwiLCJXVCI6Mn0%3D%7C3000%7C%7C%7C&sdata=pZkC9wrHJxTZlXIw9mqDqNg0aaQOfAil%2BfUUL0V4RMY%3D&reserved=0). PACE helps ensure that figures meet PLOS requirements. To use PACE, you must first register as a user. Registration is free. Then, login and navigate to the UPLOAD tab, where you will find detailed instructions on how to use the tool. If you encounter any issues or have any questions when using PACE, please email PLOS at [figures@plos.org](mailto:figures@plos.org). Please note that Supporting Information files do not need this step.

We have used the Preflight Analysis and Conversion Engine (PACE) and generated new tif-files for fig1-fig5.

When submitting your revision, we need you to address these additional requirements.

1. Please ensure that your manuscript meets PLOS ONE's style requirements, including those for file naming. The PLOS ONE style templates can be found at

<https://journals.plos.org/plosone/s/file?id=wjVg/PLOSOne_formatting_sample_main_body.pdf> and [https://journals.plos.org/plosone/s/file?id=ba62/PLOSOne_formatting_sample_title_authors_affiliations.pdf](https://eur03.safelinks.protection.outlook.com/?url=https%3A%2F%2Fjournals.plos.org%2Fplosone%2Fs%2Ffile%3Fid%3Dba62%2FPLOSOne_formatting_sample_title_authors_affiliations.pdf&data=05%7C01%7Csolveig.ose%40sintef.no%7Cebe7732f4c5542db9d3a08db26740680%7Ce1f00f39604145b0b309e0210d8b32af%7C1%7C0%7C638146052159649758%7CUnknown%7CTWFpbGZsb3d8eyJWIjoiMC4wLjAwMDAiLCJQIjoiV2luMzIiLCJBTiI6Ik1haWwiLCJXVCI6Mn0%3D%7C3000%7C%7C%7C&sdata=4YWBwN9q2sT7GJwYmN85O2ZyAY7fLwezsQB6Ae%2FUwOM%3D&reserved=0).

2. Please provide additional details regarding participant consent. In the ethics statement in the Methods and online submission information, please ensure that you have specified what type you obtained (for instance, written or verbal, and if verbal, how it was documented and witnessed). If your study included minors, state whether you obtained consent from parents or guardians. If the need for consent was waived by the ethics committee, please include this information.

Yes, this was also included originally.

3. We note that the grant information you provided in the ‘Funding Information’ and ‘Financial Disclosure’ sections do not match. When you resubmit, please ensure that you provide the correct grant numbers for the awards you received for your study in the ‘Funding Information’ section.

Yes, this is included in the Cover letter.

4. Thank you for stating the following financial disclosure:

“Funding for the data collection was provided by the Norwegian Nurses Organisation (NSF). No specific funding was received for this publication.”

Please state what role the funders took in the study.  If the funders had no role, please state: ""The funders had no role in study design, data collection and analysis, decision to publish, or preparation of the manuscript.""

If this statement is not correct you must amend it as needed.

Please include this amended Role of Funder statement in your cover letter; we will change the online submission form on your behalf.

Thank you, the funding paragraph is now included in the Cover letter.

Yes, we have now included a Funding section in the Cover letter: "Funding for the data collection was provided by the Norwegian Nurses Organisation (NSF). No specific funding was received for this publication. The funder had no role in the decision to publish, the analyses or in interpretation of results and preparation of the manuscript."

5. In your Data Availability statement, you have not specified where the minimal data set underlying the results described in your manuscript can be found. PLOS defines a study's minimal data set as the underlying data used to reach the conclusions drawn in the manuscript and any additional data required to replicate the reported study findings in their entirety. All PLOS journals require that the minimal data set be made fully available. For more information about our data policy, please see <http://journals.plos.org/plosone/s/data-availability>.

Upon re-submitting your revised manuscript, please upload your study’s minimal underlying data set as either Supporting Information files or to a stable, public repository and include the relevant URLs, DOIs, or accession numbers within your revised cover letter. For a list of acceptable repositories, please see <http://journals.plos.org/plosone/s/data-availability#loc-recommended-repositories>. Any potentially identifying patient information must be fully anonymized.

Important: If there are ethical or legal restrictions to sharing your data publicly, please explain these restrictions in detail. Please see our guidelines for more information on what we consider unacceptable restrictions to publicly sharing data: <http://journals.plos.org/plosone/s/data-availability#loc-unacceptable-data-access-restrictions>. Note that it is not acceptable for the authors to be the sole named individuals responsible for ensuring data access.

We will update your Data Availability statement to reflect the information you provide in your cover letter.

6. We note that you have indicated that data from this study are available upon request. PLOS only allows data to be available upon request if there are legal or ethical restrictions on sharing data publicly. For more information on unacceptable data access restrictions, please see <http://journals.plos.org/plosone/s/data-availability#loc-unacceptable-data-access-restrictions>.

In your revised cover letter, please address the following prompts:

a) If there are ethical or legal restrictions on sharing a de-identified data set, please explain them in detail (e.g., data contain potentially sensitive information, data are owned by a third-party organization, etc.) and who has imposed them (e.g., an ethics committee). Please also provide contact information for a data access committee, ethics committee, or other institutional body to which data requests may be sent.

b) If there are no restrictions, please upload the minimal anonymized data set necessary to replicate your study findings as either Supporting Information files or to a stable, public repository and provide us with the relevant URLs, DOIs, or accession numbers. For a list of acceptable repositories, please see <http://journals.plos.org/plosone/s/data-availability#loc-recommended-repositories>.

We will update your Data Availability statement on your behalf to reflect the information you provide.

Response: Yes, anonymous data are available through Norwegian Agency for Shared Services in Education and Research (Attribution-NonCommercial 4.0 International (CC BY-NC 4.0)). We still wait for a doi and will provide this when we receive it.

7. Please review your reference list to ensure that it is complete and correct. If you have cited papers that have been retracted, please include the rationale for doing so in the manuscript text, or remove these references and replace them with relevant current references. Any changes to the reference list should be mentioned in the rebuttal letter that accompanies your revised manuscript. If you need to cite a retracted article, indicate the article’s retracted status in the References list and also include a citation and full reference for the retraction notice.
Response: Changes in the reference list are due to comments from reviewers and is included in the response to reviewers document.
